# 

**Published:** 1864-02

**Authors:** 


*Contents.*

ORIGINAL COMMUNICATIONS.......... 17

1. Report on the Yellow Fever Epidemic at Wilmington, N. C., in the Autumn of 1862, by Surgeon Wm. T. Wragg. 2. A Case of Exomphalos, by H. Wythe Davis, M D., Richmond, Va. 3. Nelaton's Method of Detecting the Position of the Ball in Gun-Shot Wounds, with Illustrative Cases—i. Gun Shot Wound of the Thigh; Ball lodged in the Condyle of the Femur; detected by Nelaton's Probe and Canula Forceps; successful removal; by Surgeon James Bolton, Richmond, Va.—ii. Letter to the Editor from Surgeon A. Y. P. Garnett, on the Use of Nelaton's Probe. 4. Ligature of the Right Subclavian artery, reported by Surgeon P. F. Browne, Chimborazo Hospital, Division No. I.

CONFEDERATE STATES HOSPITAL REPORTS.......... 24

1. Report of Cases of Compound Comminuted Fracture of Femur, Chimborazo Hospital, Third Division, by Surgeon E. H. Smith. 2. Synopsis of the Consolidated Reports of the Hospitals in the Department of Virginia, from September, 1862, to December, 1863, inclusive, by Surgeon W. A. Carrington, Medical Director.

EDITORIAL AND MISCELLANEOUS.......... 25

Association of Army and Navy Surgeons. Resection at Hip Joint (Read's Case). The Medical Department of the Confederate States Army—its relation to the other brances of the service, and the duties of its officers.

CHRONICLE OF MEDICAL SCIENCE.......... 26

1. Remarks concerning the Hygiene of Military Hospitals, extracted from a paper read before the Imperial Academy of Medicine at Paris, by M. H. Baron Larrey; Paris, 1861; [translated from the French and abridged by Professor J. L. Cabell, University of Virginia, Surgeon P. A. C. S.] 2. Hydrocyanic Acid in the Treatment of Insanity, by Kenneth McLeod, M D. 3. French Medical News. 4. Albuminous Urine in Puerperal Convulsions. By F. H. Ramsbotham, M. D. 5. The Treatment of the Local Effects of Cold, by Krajewski, of Krubieszon.

